# Design Space Exploration of Clustered Sparsely Connected MPSoC Platforms

**DOI:** 10.3390/s22207803

**Published:** 2022-10-14

**Authors:** Nikolina Frid, Vlado Sruk, Domagoj Jakobović

**Affiliations:** Faculty of Electrical Engineering and Computing, University of Zagreb, Unska 3, 10000 Zagreb, Croatia

**Keywords:** design space exploration, heterogeneous multiprocessor systems, sparsely connected platforms, evolutionary multi-objective optimization, NSGA-II

## Abstract

Heterogeneous multiprocessor platforms are the foundation of systems that require high computational power combined with low energy consumption, like the IoT and mobile robotics. In this paper, we present five new algorithms for the design space exploration of platforms with elements grouped in clusters with very few connections in between, while these platforms have favorable electric properties and lower production costs, the limited interconnectivity and inability of heterogeneous platform elements to execute all types of tasks, significantly decrease the chance of finding a feasible mapping of application to the platform. We base the new algorithms on the Non-dominated Sorting Genetic Algorithm II (NSGA-II) meta-heuristic and the previously published SDSE mapping algorithm designed for fully interconnected multiprocessor platforms. With the aim to improve the chance of finding feasible solutions for sparsely connected platforms, we have modified the parts of the search process concerning the penalization of infeasible solutions, chromosome decoding, and mapping strategy. Due to the lack of adequate existing benchmarks, we propose our own synthetic benchmark with multiple application and platform models, which we believe can be easily extended and reused by other researchers for further studying this type of platform. The experiments show that four proposed algorithms can find feasible solutions in 100% of test cases for platforms with dedicated clusters. In the case of tile-like platforms, the same four algorithms show an average success rate of 60%, with one algorithm going up to 84%.

## 1. Introduction

Modern embedded systems targeting areas of application such as the Internet of Things (IoT), mobile robotics, or multimedia processing require high computational power combined with low energy and manufacturing costs. Multiprocessor system-on-chip (MPSoC) platforms are often the best choice because they integrate heterogeneous processing, memory, and communication elements. The most prominent product lines today are Xilinx Zynq-7000 SoC Family [1], Intel Stratix 10 FPGAs [2], and Adapteva Parallella [3]. The high degree of MPSoC platform integration requires efficient communication between platform elements to complement the processing power while, at the same time, observing printed circuit board (PCB) limitations. With the power consumption quadratic to the number of interconnections, issues with power dissipation, signal propagation, etc., can arise, and it is necessary to minimize the number of communication paths. The platform elements are very often grouped into small clusters, and the network on a chip (NoC) is used to reduce the number of connections while keeping a path between each processor. The composition of elements in clusters can vary. For example, platform elements of the same type can be grouped in homogeneous dedicated clusters, each one intended for a specific purpose: signal processing, graphics, general processing, etc., such as Xilinx Ultrascale MPSoC [4], and SI DSP board [5]. The other possibility is to have multiple identical or very similar clusters, internally heterogeneous, resembling a tile-like structure that allows easy scaling up by adding new tiles, such as the CoreVA platform [6].

On reconfigurable platforms, like FPGA, it is possible to customize communication paths and tailor them for a specific application. However, reducing the number of connections up to the point where there are no direct paths between some of the platform elements, creates a significant challenge for the design space exploration (DSE). Mapping the software application to the hardware platform is an NP-hard problem even for the fully interconnected heterogeneous platforms [7] due to the limited ability of processors to execute certain operations. Combining such a constraint with the severely reduced platform interconnectivity significantly increases the number of infeasible solutions in the design space, potentially even outnumbering feasible ones. Consequently, this increases the chance that the search algorithm can have trouble converging and ultimately fail never reaching a feasible solution [8], especially when using common evolutionary algorithms [9,10].

A possible solution to this issue is to adapt existing DSE methods designed for MPSoC platforms, which can already cope with the constraints like the ability of a processor to execute a specific type of task. The Single-phased Design Space Exploration (SDSE) algorithm, published in [11], is a mapping algorithm designed for heterogeneous MPSoC platforms and employs the NSGA-II evolutionary meta-heuristic for searching the design space. It is 100% successful in finding feasible solutions on fully connected platforms but lacks the support for sparsely connected platforms. In this paper, we extend and modify the SDSE algorithm, and propose five new design space exploration algorithms with the aim of increasing the probability of finding feasible solutions for sparsely connected MPSoC platforms. To facilitate pruning infeasible solutions early and efficiently, we modify the search process by changing the chromosome decoding procedure, task-to-processor mapping procedure, and penalization of infeasible solutions.

To test these new algorithms, and because of the lack of adequate existing benchmarks due to the nature of this specific problem, we propose a new synthetic benchmark with multiple application and platform models. The benchmark contains six platform models that represent different types and layouts of the clusters. There are also 28 application models with a different number of task predecessors and successors, the ratio of communication to computation, and the suitability of tasks for execution on different processor types. We have tested all the algorithms using this benchmark and presented the results in detail. In brief, four proposed algorithms found feasible solutions in 100% of test cases for the platforms with dedicated clusters, which is a significant improvement compared to the SDSE algorithm that was successful in only 4% of test cases. In the case of tile-like platforms, the average success rate for the same four algorithms is around 60%, but one algorithm had an average success rate of 84%, with up to 100% for some layouts, and hence shows promising results for future advancement.

The main contributions of this paper can be summarized as follows:

- Five multi-objective algorithms for design space exploration of sparsely connected heterogeneous clustered multiprocessor platforms are proposed:
  – Algorithms are based on the NSGA-II meta-heuristic, and each one of them deals with infeasible solutions in the design space by using different strategies for mapping application to platform, as well as penalization of infeasible solutions.
  – Four of these algorithms are inherently designed for cluster-based platforms, and perform task mapping in two stages: tasks are first mapped to clusters, and then each task is mapped to one of the processors inside the cluster.
- A new custom synthetic benchmark, with multiple high-level models of various application and platform architectures, is proposed for the evaluation of the considered algorithms. The models in the benchmark are designed to have the same hard constraints as real-world heterogeneous MPSoCs.
  – Platform models include specialized types of processors that can execute only certain types of tasks.
  – Processing and memory elements are grouped in clusters, and only one processor from each cluster can be used for communication with other clusters.
  – Application models incorporate task concurrency and precedence constraints.
  – Models can be modified to increase the number of clusters and/or the size of the cluster.
- Extensive evaluation of the proposed algorithms on over 150 test cases.

The paper is organized as follows. In Section 2, work related to the design space exploration of heterogeneous MPSoC systems is reviewed. The problem definition is given in Section 3. Section 4 provides the required background knowledge related to multi-objective optimization, and presents the new DSE algorithms. The synthetic benchmark used for evaluation is presented in Section 5, followed by a discussion of experimental results. Section 6 concludes the paper and discusses future work.

## 2. Related Work

The goal of design space exploration is to find an optimal mapping of an application to a platform. For heterogeneous MPSoC platforms, it is an NP-hard problem with no guarantee of finding the optimal solution in a reasonable amount of time for all but the most trivial problem instances. To deal with this complexity, most of the existing design space exploration methods for MPSoC systems rely on System Level Design (SLD) principles [12] and use separate application and platform models at a high level of abstraction. SLD is the prevalent embedded systems design methodology today [13], and hence we also base our work on this principle. The downside of this approach is that relying on abstract models increases performance estimation error to around 10% [14,15], This approach enables pruning the vast design space quickly and narrowing down the list of possible candidates to only a few near-optimal solutions. The only drawback is that relying on abstract models increases performance estimation error to around 10% [14,15], and hence the few final candidates usually need to be implemented as prototypes and evaluated more thoroughly. Two of the most prominent academic DSE tools also follow the SLD approach: *Daedalus* [16] and *MAPS* [17]. However, these tools do not take into account the existence of multiple communication paths between processors (i.e., multiple different memories to which processors can be connected and exchange data, and so the memory configuration and data allocation scheme are determined manually at a later stage of design and implementation.

In other recent works, which also consider memory configuration, both deterministic and meta-heuristic approaches are used to solve the task mapping problem. Integer linear programming (ILP) is a popular example of a deterministic strategy, used in [8,15,18,19]. A comprehensive approach, focusing on integrating memory allocation into the DSE, is found in the following papers. Salamy and Ramanujam [20] present a heuristic that integrates task scheduling and memory partitioning for applications on heterogeneous MPSoC platforms with scratchpad memory. However, the authors consider a very simple memory architecture with one scratchpad memory (SPM) split between two processors and one main working memory. Jovanovic et al. [21] consider heterogeneous MPSoC systems with different types of processors and memories, but memory allocation occurs after assigning tasks to processors, and their work does not consider the possibility of simultaneous evaluation of processor-memory pairs. Although the authors claim an improvement of about 18% for standard test cases (FIR, IIR, MPEG4, compression, etc.), the optimization method is based on ILP, which means that for more complex problems, the time to arrive at a solution is very long and possibly infinite. Goens et al. [19] use Mixed Integer Programming (MILP) to optimize memory allocation on a heterogeneous platform. Again, the data allocation scheme in memory is generated after assigning tasks to processors. The method is focused primarily on streaming applications, so the goal of optimization is not to find the optimal solution, but only the one that meets the given constraints. This relaxation of the problem shortens the execution time of the MILP, but for the larger memory space, according to the authors, the proposed method did not find solutions even after running for four days on a supercomputer.

Since the time complexity of ILP-based algorithms does not allow them to scale well to larger problem sizes, meta-heuristic algorithms, such as evolutionary and swarm algorithms, are more commonly used today [22]. They do not make assumptions on the objective functions, and because of that are more flexible, efficient and can find near-optimal solutions in a considerably shorter time [16,23,24,25,26,27]. Typically, these algorithms execute many unguided and independent searches from different starting points to increase the chance of reaching global optima.

More recent works also emphasize the need for more efficient use of limited resources in multiprocessor systems with distributed nodes. Authors in both [28,29] focus on Wireless Sensor Networks (WSN) widely used today in many areas, such as agriculture. Because WSNs are composed of nodes with limited processing, memory, and battery resources, authors suggest minimizing communication to save energy and improve overall performance. By using clustering techniques to select cluster heads (CH) as the nodes that will connect nearby nodes with the base station, they are able to reduce power consumption. They demonstrate that a combination of fuzzy-based heuristics and meta-heuristic algorithms: Shuffled Frog Leaping Algorithm (SFLA) and Fuzzy Firefly algorithm combined with Random Forest, respectively, are able to find high-quality solutions at higher speeds than classical meta-heuristics alone. In [30], the authors also recognize the impact of the interconnect architecture on the performance of a multiprocessor system, area, power consumption, and signal properties, and outline the necessity to balance performance, reliability, and resource usage. The paper proposes a reliability-aware task mapping technique to deal with permanent failures that can occur during run-time on MPSoCs, and require migrating tasks to other operational processing elements. They propose a fault-tolerant technique based on a combination of a multi-objective version of the Biogeography-Based Optimization Algorithm (MOBBO), and reinforcement learning (RL) using Deep Q-learning (DQL). However, all three papers consider only homogeneous systems, and thus it is unknown how the proposed methods would perform in heterogeneous environments.

To the best of our knowledge, none of the published works consider the DSE problem for heterogeneous platforms on which processing elements are not fully interconnected either via NoC, wireless network, or in another similar manner. In such cases, the hard constraints regarding the ability to execute certain types of tasks, and platform interconnectivity, make the DSE process challenging by creating a large space of combinations, of which only a small fraction represent feasible solutions. For such systems, it is important to consider processor and memory mapping simultaneously to be able to properly recognize infeasible solutions. In our previous work [11], we developed a DSE method for heterogeneous multiprocessor platforms named Single-phase Design Space Exploration (SDSE) algorithm that can simultaneously map computation to processing and communication to memory elements, while optimizing two objectives simultaneously: total execution time and the number of platform elements used. It can also successfully handle typical MPSoC mapping constraints like the order of task execution, and the inability of certain types of processors to execute certain types of tasks. It is based on the evolutionary meta-heuristic NSGA-II algorithm, which gives it flexibility and makes it easily adaptable to different platform setups. However, this method is not designed and has never been tested, for platforms with sparse connectivity, for example where each processor is directly connected only to a small sub-group of processors, etc. Furthermore, while it should be able to perform the DSE on such configurations, there are no guarantees that a feasible solution will be found. This paper is a continuation of the previous work with the aim to develop a DSE method more suitable for sparsely connected MPSoC platforms, namely clustered-based architectures. We keep the SLD approach to system modeling and the NSGA-II meta-heuristic, but modify the search process of the original SDSE algorithm by changing the chromosome decoding procedure, task-to-processor mapping procedure, and penalization of infeasible solutions. The main contribution building on the existing work is adapting the evolutionary meta-heuristics approach to multiprocessor architectures with sparse connectivity, and heterogeneous composition of platform elements.

## 3. Problem Definition

Design space exploration is defined as the problem of finding a near-optimal mapping of an application to a platform. As required by the SLD methodology, the system is modeled at a high level of abstraction using separate architecture and application models to decouple communication from computation. The application is modeled as a set of tasks connected via communication channels in a directed acyclic graph (DAG). The platform is modeled as a set of processing and memory elements: processors are connected to memories and exchange data by writing and reading data from memory elements. The mapping of the application to the platform is defined as a two-criteria optimization problem, the goal of which is to minimize both the total execution time and the number of platform elements used. A detailed description of the application model, platform model, and the optimization problem is described as follows.

### 3.1. Application Model

**Figure 1:**
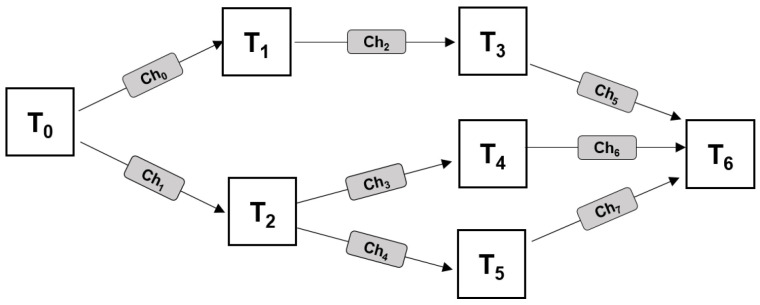
An example application model with seven tasks and eight communication channels depicted using a directed acyclic graph (DAG).

The application consists of one or more tasks that execute in sequence or in parallel. There is always only one task at the start and one at the end, but in between each task can have one or more predecessors and one or more successors. A task can start executing after all of its predecessors have finished. The flow of data between tasks is directed from predecessors to successors. Thus, a directed acyclic graph (DAG) is used as the application model. Nodes in the graph represent tasks, and directed edges between the nodes represent communication channels. Communication takes place only between procedures that are directly connected in the DAG, in the direction of the connection. Edge weight represents the amount of data that is sent over the channel. Figure 1 illustrates an example of a generic application consisting of several procedures interconnected by communication channels.

### 3.2. Platform Model

The platform model is a graph with two types of nodes: processing and memory. The processing and memory elements are the most important elements of any hardware platform, and their characteristics have the highest impact on system performance. Processing nodes execute tasks from the application model, and for each combination of task and processor, the execution time is defined. Processors are connected to memory nodes through which they exchange data. Memory nodes have a defined size and number of ports. Ports can support read, write, or both operations. Edges in the graph represent links between processing and memory elements. For each connection, reading and writing speed are defined. In this model, memory elements are used solely for communication between processors, while the instruction and data storage memory for each processor is considered predefined and immutable, i.e., an inherent part of the processing node. Details related to cache memory, operating system, communication protocols, etc., are omitted in these idealized models.

**Figure 2:**
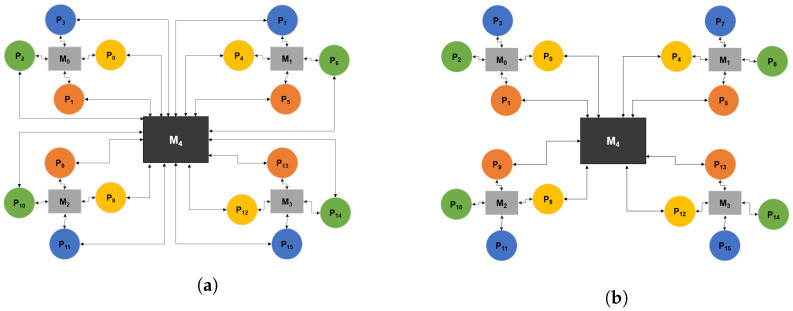
Two examples of platform models. (**a**) depicts a fully interconnected platform, and (**b**) depicts a sparsely interconnected platform.

Figure 2 illustrates two examples of platform models. The first one shows a fully interconnected platform model in which there is a connection between each pair of processors. The second one is an example of a sparsely connected platform on which processing elements are grouped in small clusters around one memory element, and the larger memory element serves as a hub between clusters to which only a few processors from each cluster are connected.

### 3.3. Optimization Problem

The goal of design space exploration is to find an optimal or near-optimal mapping of an application to a platform. If the application is defined as a set of tasks and communication channels APP=(T,Ch), and the platform is defined as a set of processing and memory elements and links between them PL=(P,M,L), then tasks are mapped to processors and communication channels are mapped to memories.

(1) $$\left(\mu_{t}:T\to P,\mu_{ch}:Ch\to M\right)$$

A mapping scheme is considered to be a feasible solution only if it satisfies the following two constraints: (1) a task must be mapped to a processor that can execute all its operations, and (2) consecutive tasks must be mapped to processors which are connected to the same memory. The first constraint is typical for heterogeneous MPSoC systems in general, where it is possible to have hardware cores specialized only for a specific operation (e.g., DCT image transformation), and which cannot execute any other operations. The second constraint arises from the platform topology: on a sparsely connected platform, there is no guaranteed connection between each possible pair of processors.

An optimal solution, in this case, is a feasible mapping scheme that minimizes the total execution time ($C_{max}$) and the total number of platform elements used ($N_{max}$). The $C_{max}$ objective is the time when the last task has finished its execution. The $N_{max}$ objective is calculated by counting the processing and memory elements that have at least one task, or communication channel mapped to them. If an element is used twice, or more, (e.g., a processor has three tasks mapped to it), it will still contribute to the $N_{max}$ objective with +1. The rationale behind the design of the $N_{max}$ objective is that in the case when the final design is synthesized in FPGA or ASIC, it is not necessary to use all the platform elements given in the initial model, but only the ones that are used will be synthesized. In such a case, using a smaller number of elements will usually lower the final product cost.

To define the optimization problem, we introduce the following notation:

*T*: total number of tasks
*Ch*: total number of communication channels
*P*: total number of processors
*M*: total number of memories
*i*: task index, *i* = 0, 1, 2, …, *T* − 1
*j*: communication channel index, *j* = 0, 1, 2, …, *Ch* − 1
*k*: processor index, *p* = 0, 1, 2, …, *P* − 1
*l*: memory index, *i* = 0, 1, 2, …, *M* − 1
$C_{i}$: completion time of task *i*

Furthermore, we use decision variables to define whether a certain task is mapped to a processor, or a communication channel is mapped to a memory:

(2) $$X_{ik}=\left\{\begin{array}{cc} 1 & \mathrm{when}\;\mathrm{task}\;i\;\mathrm{is}\;\mathrm{mapped}\;\mathrm{to}\;\mathrm{processor}\;k\\ 0 & \mathrm{otherwise} \end{array}\right.$$

(3) $$Y_{jl}=\left\{\begin{array}{cc} 1 & \mathrm{when}\;\mathrm{communication}\;\mathrm{channel}\;j\;\mathrm{is}\;\mathrm{mapped}\;\mathrm{to}\;\mathrm{memory}\;l\\ 0 & \mathrm{otherwise} \end{array}\right.$$

Finally, the optimization problem is defined as:

(4) $$min(C_{max},N_{max}),$$

with two objectives $C_{max}$ and $N_{max}$ defined as:

(5) $$C_{max}=max_{i=0,1,\ldots ,T-1}\left(C_{i}\right)$$

(6) $$N_{max}=\sum_{k=0}^{P-1}min\left(1,\sum_{i=0}^{T-1}X_{ik}\right)+\sum_{l=0}^{M-1}min\left(1,\sum_{j=0}^{Ch-1}Y_{jl}\right)$$

## 4. Methodology

For solving this multi-objective optimization problem, we propose five new mapping algorithms, all of which use the NSGA-II multi-objective meta-heuristic [31] for searching the design space. These algorithms have been developed from the SDSE algorithm, with which they share the chromosome structure and scheduling techniques. The novel contribution of this work is the introduction of new application-to-platform mapping strategies which aim to manage additional constraints that appear in sparsely connected platforms (i.e., there does not exist a connection between every pair of processors), and direct the search process to prune infeasible solutions more effectively.

In the rest of this section, we first briefly present the basic concepts of NSGA-II, along with the mapping and scheduling strategy of the SDSE algorithm. In the second subsection, we present each new algorithm in detail.

### 4.1. Background in Multi-Objective Optimization

The NSGA-II evolutionary meta-heuristic has the following three characteristics: uses elitism, uses a mechanism to explicitly preserve the diversity of solutions, and emphasizes non-dominated (Pareto optimal) solutions. In multi-objective problems, the optimum is not represented by a single solution but a set of trade-off solutions, known as the Pareto-optimal front (POF). All solutions in a population are sorted into a number of disjoint subsets or non-dominated fronts, by means of comparing their values of the same objective. Solutions in the same Pareto front are viewed as equally important. However, they can be further distinguished by additional criteria, such as the crowding distance. The crowding distance $d_{i}$ for the solution *i* is defined as a measure of the design space around *i* that is not occupied by some other solution from the population.

In the NSGA-II, in each generation *t*, the offspring population $Q_{t}$ is created from the parent population $P_{t}$ using the crossover and mutation genetic operators. Candidates for the crossover are selected using the classic 2-tournament selection (k=2), based on the non-dominated fronts to which they belong, and the crowding distance.

Subsequently, these two populations are merged to form a new population $R_{t}$ of size 2N. Then, the population $R_{t}$ is divided into fronts by performing the non-dominated sort, and the population that is passed on to the next generation $P_{t+1}$ is filled with solutions from the non-dominated fronts of the population $R_{t}$, one by one. Filling begins with the first non-dominated front, then the second, etc. Because the size of population $R_{t}$ is double that of population $P_{t+1}$, not all solutions can be moved to a new population, and solutions that do not fit are deleted. Furthermore, it is possible that all solutions from the last non-dominated front that is passed from population $R_{t}$ to population $P_{t+1}$ cannot fit. Then instead of randomly, the solutions that move to the next generation are chosen according to diversity, i.e., the crowding distance sorting of solutions in descending order is performed. The solutions from the top of the sorted list (the ones with maximum crowding distance) pass on to the next generation and the others are discarded.

There are numerous metrics in the literature to compare Pareto-optimal fronts obtained by multi-objective algorithms [32]. However, since all optimization methods are not equally effective for all types of problems [33], it is recommended to use at least as many measures as the number of optimization criteria, including metrics that measure the convergence and diversity of the solution [34]. In our work, we have selected two common metrics for comparison of solutions obtained by different algorithms: Hypervolume (HV) and Inverted Generational Distance (IGD) [32,35,36]. These metrics focus on two aspects of the quality of an approximation, i.e., convergence and diversity. Convergence is related to the closeness of an approximation to the true POF, whereas diversity in general refers to the uniformity and extensity of the approximation.

**Figure 3:**
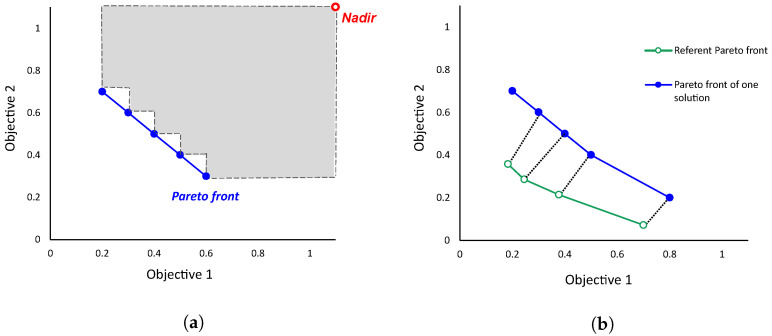
Illustration of the hypervolume (HV)—(**a**), and the inverted generational distance (IGD)—(**b**) metrics.

The HV metric is calculated as the volume of hyperspace between the algorithm’s POF and the nadir point. The nadir point is obtained by selecting the worst solution from the set of all solutions (unified solutions of all the methods being compared) and adding a *delta* to the extremes so that the endpoints on the front have some contribution to the computation of HV. When comparing the two methods, one with a higher HV is considered better. For an optimization problem with two objectives, the HV is the quadratic surface area between points in the POF and Nadir, indicated in gray in Figure 3a.

To calculate the IGD metric, first, a reference POF must be determined. This is done by putting together all solutions obtained by all algorithms, and then extracting a set of Pareto-optimal ones. Then, for solutions generated by each algorithm, the Pareto front of its own solutions is determined. Furthermore, finally, the IGD metric for an algorithm is calculated as the average value of the distance from each point of the reference Pareto front to the nearest point in the algorithm’s Pareto front, as illustrated in Figure 3b.

For implementation, we rely on the MOEA framework [37]. It is an open-source library (LGPL v3) written in Java to develop optimization algorithms. It has a basic implementation of many genetic algorithms, including NSGA-II, and the framework provides extensions and modifications as needed.

### 4.2. Mapping and Scheduling Strategy of the SDSE Algorithm

In order to apply NSGA-II for the DSE of heterogeneous MPSoCs, it is required to define an appropriate scheme for solution representation and evaluation. In the SDSE algorithm, the evaluation of the solutions is a process performed in three stages:

1. mapping tasks to processors and communication channels to memory
2. scheduling
3. calculation of two objectives: $C_{max}$ and $N_{max}$

**Figure 4:**
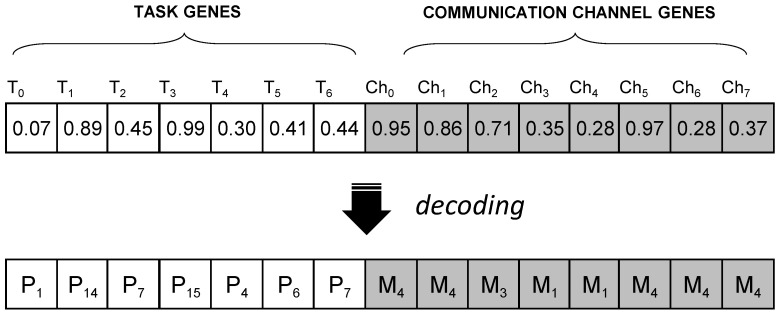
The structure of the chromosome for the application model with seven tasks and eight communication channels, and an example of how gene values are decoded to processor and memory id numbers.

In the first stage, the application is mapped to the platform: tasks are mapped to processors, and then communication channels are mapped to memories, as illustrated in the pseudocode in Algorithm  1. Task mapping starts with forming a list of eligible processors for each task (code line 2). A processor is considered eligible for a task if it can execute it. The list of eligible processors is determined based on the platform specification, described in Section 3.2. Once the list is formed, one processor is selected based on the value of the corresponding gene in the chromosome. The chromosome is defined as a composition of two arrays of genes encoded as real values. If the application model consists of *M* tasks and *N* communication channels, the chromosome will have M+N genes. The first *M* genes determine the mappings of tasks to processors, and the remaining *N* genes determine mappings of communication channels to memories. In order to obtain a mapping, the real values of each gene, in the range from 0 to 1, must be decoded to an integer number that corresponds to an existing processor or memory element. If there are *K* eligible processors, the gene value is decoded by dividing the [0, 1] interval into *K* sub-intervals of equal size so that each processor belongs to one interval. The processor that belongs to the interval which contains the gene value will be selected (line 4), and the task will be mapped to it (lines 5). The same applies to mapping communication channels to memories, which is performed after task mapping, (lines 8–14). An example of the chromosome structure for a mapping of the application from Figure 1 to the platform from Figure 2a is illustrated in Figure 4. In this example, there are 7 tasks and 8 communication channels that can be mapped to 16 processors and 5 memories, respectively. If processors $P_{1}$ to $P_{6}$ are eligible for task $T_{0}$, the interval [0, 1] will be divided into 6 sub-intervals. Since the corresponding gene value in this example is 0.07, a value from the first sub-interval, task 0 will be mapped to $P_{1}$, etc. In the same manner, if there are four existing paths between $P_{1}$ and $P_{14}$ (according to the platform model from Figure 2a), to map the communication channel $Ch_{0}$, which connects tasks $T_{0}$ and $T_{1}$, the interval [0, 1] will be divided into five sub-intervals. The corresponding gene value 0.95 belongs to the fifth sub-interval, so memory $M_{4}$ is selected.

A communication channel can only be mapped to a memory that connects processors on which the tasks connected by this channel are executed. If there are no eligible processors or memories, the mapping is infeasible and the value of both objectives is set to infinity (lines 7 and 16).
**Algorithm 1** SDSE: Mapping application to platform.  1:**for** each task **do**  2:      form a list of eligible processors  3:      **if** list not empty **then**  4:           select a processor from the list based on the gene value  5:           map task to processor  6:      **else**  7:           set objective values to infinity and exit  8:      **end if**  9:**end for**10:**for** each communication channel **do**11:      form a list of eligible memories12:      **if** list not empty **then**13:           select a memory from the list based on the gene value14:           map communication channel to memory15:      **else**16:           set objective values to infinity and exit17:      **end if**18:**end for**

If the mapping scheme is feasible, scheduling is performed using time slots, as described in [11]. In brief, a time slot represents a period of time during which a task communicates with its predecessors and successors, or is being executed on a processor. The distinction is made between communication slots used for exchanging data between a task and its predecessors/successors, and processing slots which represent the time that tasks spend executing on a processor. The goal of scheduling is to find free processing and communication slots at the earliest time possible. In the case of a fully connected platform, finding a feasible mapping and scheduling scheme is guaranteed. However, in the case of a sparsely connected platform with very specialized processors, infeasible solutions can greatly exceed the number of feasible solutions, which creates a non-negligible probability that the search algorithm will never be able to find a feasible solution.

### 4.3. Proposed Algorithms for Sparsely Connected Platforms

In the rest of this section, the proposed new algorithms are described in detail. They all retain the same NSGA-II evolutionary core, use the same chromosome structure, and apply the same scheduling technique as the SDSE, but use new mapping strategies for coping with infeasible solutions as the consequence of sparse interconnectivity. The main two avenues of exploration are (1) differentiation among infeasible solutions, and (2) adapting to the platform topology by relying on simple cluster-detection heuristics. The source code is publicly available on the Git repository at https://gitlab.com/Frid/dsesparse (accessed on 10 October 2022).

#### 4.3.1. N-SDSE Algorithm Description

In the SDSE algorithm, all infeasible solutions are treated equally, i.e., there is no distinction between an infeasible solution with only a few instances of consecutive procedures mapped to unconnected processors and an infeasible solution with many such instances. The N-SDSE (*Non-Infinity SDSE*) algorithm introduces a penalty with the aim to achieve the gradation of “bad” solutions, which is one of the most popular constraint-handling techniques [38,39]. The execution time of infeasible solutions is no longer infinite, but each time it is impossible to find a processor that can execute a task, or there is no connection between processors that execute two tasks, a large penalty is added to the total execution time. This should allow the solutions that have fewer infeasible connections, and can potentially become viable solutions through several crossovers and/or mutations, to get a higher chance to be carried to the next generation than solutions with much larger penalties (more infeasible mappings). The pseudocode for mapping is given in Algorithm 2. The difference from the SDSE algorithm is that in case there are no eligible processors or memories, the task or communication channel is marked as unmapped (lines 7 and 16), but the mapping process continues. After the mapping is finished, the scheduling is carried out using the same time slot method as in the SDSE. However, in the case when some tasks and/or channels have been marked as unmapped, the time slots assigned to them are each set to last $10^{10}$, which is the value of the penalty. This particular value of penalty, which is around 10 orders of magnitude larger than task execution times, has been determined through trial and error: penalty values significantly lower (by several orders of magnitude) were not able to produce feasible solutions at all, and penalties several orders of magnitude higher showed the same behavior as the chosen value.
**Algorithm 2** N-SDSE: Mapping application to platform.  1:**for** each task **do**  2:      form a list of eligible processors  3:      **if** list not empty **then**  4:           select a processor from the list based on the gene value  5:           map task to processor  6:      **else**  7:           mark task as unmapped  8:      **end if**  9:**end for**10:**for** each communication channel **do**11:      form a list of eligible memories12:      **if** list not empty **then**13:           select a memory from the list based on the gene value14:           map communication channel to memory15:      **else**16:           mark channel as unmapped17:      **end if**18:**end for**

The time complexity for the mapping in the N-SDSE algorithm is the same as for the SDSE algorithm and is O(T∗P+Ch∗M), where *T*, *P*, Ch, and *M* represent the number of tasks, processors, communication channels, and memory elements, respectively. Here, it is worth mentioning that forming the list of eligible processors and memories (code lines 2 and 11) can be executed in parallel, in which case the time complexity can be reduced to O(T+Ch) if the number of CPU cores on the experimental computer is greater than *P* and *M*.

#### 4.3.2. C-SDSE Algorithm Description

The C-SDSE (*Clustered SDSE*) algorithm introduces a modification specifically targeting sparsely connected clustered platforms. The main idea is to do task mapping in two stages: first map tasks to clusters, and then map each task to one of the processors inside the (previously assigned) cluster.

**Figure 5:**
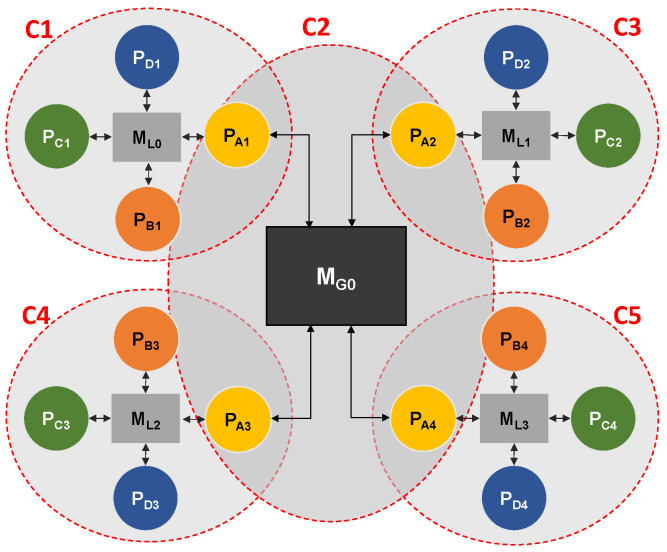
An example of cluster detection for the C-SDSE algorithm.

The detection of clusters on the platform is done beforehand following a simple heuristic: any group of processors connected to the same memory is considered a cluster. An example of cluster detection for platform model from Figure 2b illustrated in Figure 5. In the figure, clusters are marked as “C1”, “C2”, etc. As shown in the illustration, in the case when a processor is connected to more than one memory element, it will be associated with more than one cluster.

Mapping tasks to clusters starts with forming a list of eligible clusters for each task. The cluster is considered to be eligible only if it contains at least one processor that can execute the task. Afterward, the cluster is selected based on the value of the corresponding gene in the chromosome (genes that represent tasks). The decoding of the real number value stored in the gene is done in the same way as described before. Lines 2–6 in the pseudocode in Algorithm 3 summarize the process. In the second step, an attempt at mapping the task to a processor inside the previously assigned cluster is made (lines 7–17). Again, a list of eligible processors is created first, and then a processor is selected from that list based on the value of the corresponding gene. Determining the list of eligible processors involves looking also at the mappings of succeeding tasks. Only processors that can execute the task and are located in both clusters (the one to which the task belongs and the one to which its successor belongs) are eligible. In the case of a sparsely connected platform, it is possible to encounter infeasible solutions, which means mapping will fail (line 14) and objectives are then set to infinity. If the platform is fully connected (regardless of the layout), finding a feasible solution is guaranteed. In the case of a task with multiple successors, the algorithm will select the processor according to the task’s last successor, which can potentially cause problems in finding feasible solutions on sparsely connected platforms. However, there is no straightforward solution to this issue since each task can have multiple predecessors and multiple successors (N-to-N) and different approaches are possible. We have addressed this situation, by proposing a different mapping strategy for the algorithm in Section 4.3.4. The final step is mapping communication channels to memories (lines 18–26). For each communication channel, first, a list of candidate memories is formed. A communication channel can only be mapped to a memory that connects processors on which the tasks connected by this channel are executed. Here, it is also possible to have infeasible solutions.

The time complexity for the mapping in the C-SDSE algorithm is $O(T^{2}*P+Ch^{2}*M)$. This algorithm is more complex compared to SDSE and N-SDSE because determining the list of eligible processors for each task involves considering the mappings of succeeding tasks. Furthermore, in the same manner as for the N-SDSE, forming the list of eligible clusters, processors and memories can be executed in parallel, in which case the time complexity can be reduced to $O(T^{2}+Ch^{2})$ if the number of CPU cores on the experimental computer is greater than *P* and *M*.
**Algorithm 3** C-SDSE: Mapping Application to Platform.  1:detect clusters  2:**for** each task **do**  3:      form a list of eligible clusters  4:      select a cluster from the list based on the gene value  5:      map task to the selected cluster  6:**end for**  7:**for** each task **do**  8:      **for** each successor of this task **do**  9:            form a list of eligible processors10:            **if** the list is not empty **then**11:                 select a processor from the list based on the gene value12:                 map task to processor13:            **else**14:                 set objective values to infinity and exit15:            **end if**16:      **end for**17:**end for**18:**for** each communication channel **do**19:      form a list of eligible memories20:      **if** the list is not empty **then**21:            select a memory from the list based on the gene value22:            map communication channel to memory23:      **else**24:            set objective values to infinity and exit25:      **end if**26:**end for**

#### 4.3.3. CN-SDSE Algorithm Description

The CN-SDSE (*Clustered Non-Infinity SDSE*) algorithm is a modification of C-SDSE, that penalizes infeasible solutions based on the number of consecutive tasks that are mapped to unconnected processors. In the same way as it is done in the N-SDSE algorithm, in the case when there are no eligible processors or memories, the task or communication channel will be marked as unmapped, but the mapping process will continue. After the mapping is finished, and during the scheduling in the case when some tasks and/or channels have been marked as unmapped, the time slots assigned to them will be set to last $10^{10}$, which is the value of the penalty. The time complexity for the CN-SDSE is the same as for the C-SDSE.

#### 4.3.4. MC-SDSE Algorithm Description

In the case when an application is composed of tasks that have multiple predecessors and successors, the C-SDSE mapping algorithm will eventually encounter a situation where consecutive tasks will be mapped to processors in different clusters that are not connected to the same memory, which represents an infeasible solution. To try and overcome this issue, we propose a modified mapping strategy, the MC-SDSE (*Main Cluster SDSE*) algorithm, which gives priority to processors connected to a memory element that serves as a connection point (hub) between clusters. An example of such connection point is illustrated in Figure 2b in Section 3.

Before the mapping process, it is necessary to detect all clusters (as in C-SDSE), and identify the cluster around the main hub, named “main_cluster” in pseudocode in Algorithm 4 (lines 1–2). In Figure 5, cluster “C2” would be identified as the main cluster. Task mapping is done in two iterations. In the first iteration, each task is mapped to a cluster that contains at least one processor that is both connected to the main cluster and that can execute the task (lines 4–6). If there are no such clusters, then the task is mapped to a cluster that contains at least one processor that can execute that task. In the second iteration, each task is mapped to a processor, meeting the same criteria (lines 7–9). As a result, after this iteration, most tasks are going to be mapped to processors that are connected to the main cluster, while other processors will remain unused. In the second iteration, to even the load distribution, an attempt at remapping each task is carried out in the second iteration (lines 8–14), but only under the condition that all of the task’s predecessors and successors were initially mapped to the same cluster (line 6). Otherwise, it would potentially lead to an infeasible solution. Finally, communication channels are mapped to memories (lines 18–26), in the same manner as in the C-SDSE algorithm.

This approach will guarantee a feasible solution for all cases, except for the case when the application contains tasks that cannot be executed on any of the processors in the main cluster. However, even in that case, depending on the distribution of tasks predecessors and successors across clusters, finding a feasible solution is possible.
**Algorithm 4** MC-SDSE: Mapping Application to Platform.  1:detect clusters  2:identify main_cluster  3:**for** each task **do**  4:      form a list of candidate clusters  5:      select a cluster from the list based on the gene value  6:      map task to the selected cluster  7:      form a list of eligible processors  8:      select a processor from the list based on the gene value  9:      map task to the selected processor10:**end for**11:**for** each task **do**12:      **if** all task predecessors and successors are on the same cluster **then**13:           form a list of eligible processors14:           select a processor from the list based on the gene value15:           remap task to the selected processor16:      **end if**17:**end for**18:**for** each communication channel **do**19:      form a list of eligible memories20:      **if** the list is not empty **then**21:           select a memory from the list based on the gene value22:           map communication channel to memory23:      **else**24:           set objective values to infinity and exit25:      **end if**26:**end for**

The time complexity for the mapping in the MC-SDSE algorithm is the same as for C-SDSE because determining the list of eligible processors for each task involves looking also at the mappings of preceding and succeeding tasks.

#### 4.3.5. MCN-SDSE Algorithm Description

MCN-SDSE (*Main Cluster Non-Infinity SDSE*) algorithm introduces gradation of infeasible solutions to the MC-SDSE in the same way as it is done in the N-SDSE algorithm. In the case when there are no eligible memories, the communication channel will be marked as unmapped, but the mapping process will continue. After the mapping is finished, and during the scheduling in the case when some communication channels have been marked as unmapped, the time slots assigned to them will be set to last $10^{10}$, the value of penalty. The time complexity for the MCN-SDSE is the same as for the MC-SDSE.

## 5. Evaluation

The proposed DSE algorithms have been evaluated using a set of high-level application and platform models. Depending on their purpose, multiprocessor embedded systems can have numerous variations in platform architecture, and thus it is difficult to define standardized tests, so it is not uncommon that different authors, like [40,41] propose a custom benchmark tailored towards the specific architecture they explore in their work. In this paper, we focus on sparsely connected heterogeneous multiprocessor platforms with processing elements grouped in clusters, for which there is a lack of appropriate high-level benchmark models, and hence we propose our benchmark for testing DSE methods. This benchmark features high-level models of different application and platform architectures. Platform models are designed to represent various layouts of heterogeneous multiprocessor platforms with processing elements grouped in clusters, inside of which processors are fully interconnected, but connections between the clusters are sparse. Furthermore, our platform models also feature specialized types of processors that can be used only for certain types of tasks, which is a typical property of MPSoC systems. Application models are designed to incorporate task concurrency and precedence constraints. In the rest of this section, the benchmark is described in detail, and afterward, the results of the evaluation of the proposed DSE algorithm on this benchmark are presented.

### 5.1. Benchmark

The PSPLib library for resource-constrained scheduling [42] is used as the starting point for building application and platform models. Since it already contains application models in the form of an acyclic task graph with varying degrees of concurrency, precedence constraints, and does not allow preemption, upgrading those models to the ones described in this paper only requires adding communication parameters. Furthermore, the problems in this library are grouped according to four levels of task graph network complexity and resource demand. These parameters can, in our models, be easily converted to the number of task predecessors and successors, and the ability of certain tasks to be executed on various types of processors, respectively. In the end, four PSPlib models *j301_1, j305_4, j3010_1, j3014_4*, from the *j30.sm* subset of problems, have been chosen as the basis for creating application and platform models. All of them consist of 30 tasks, and the maximum number of tasks performed in parallel varies from 7 to 10, without loops. The complexity of predecessor-successor connections and resource utilization are the lowest in j301_1, and the highest in j3014_4.

**Figure 6:**
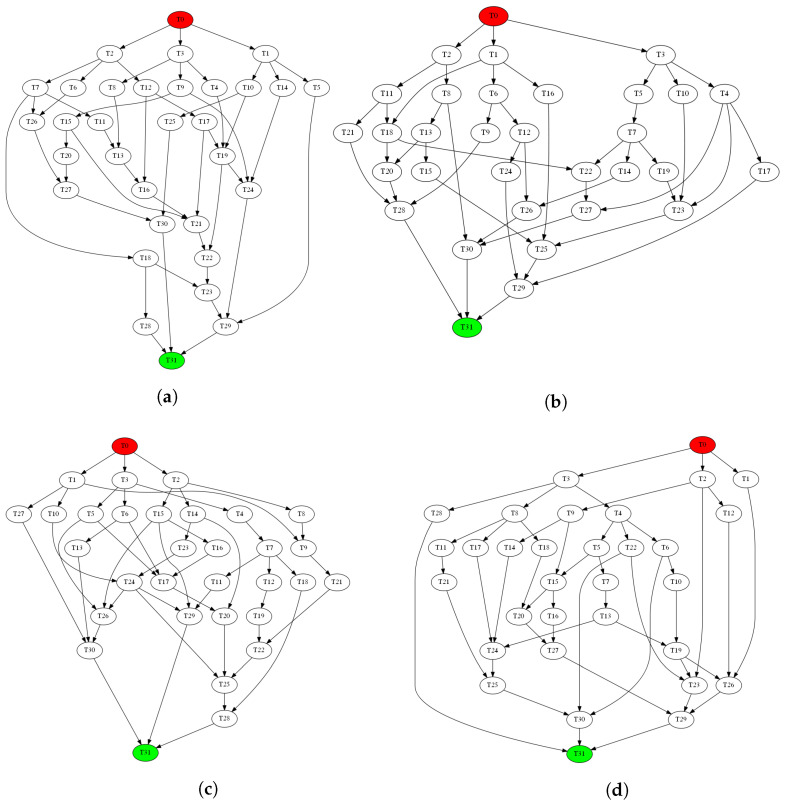
Task graphs for all four application models. (**a**) A1 (j301_1); (**b**) A2 (j305_4); (**c**) A3 (j3010_1); (**d**) A4 (j3014_4).

The application model, as described in Section 3.1, consists of a task graph (DAG) and a communication model. Task graphs of PSPLib models j301_1 to j3014_4, shown in Figure 6, are used to create four application models: *A1*, *A2*, *A3* and *A4*. To complete these models, communication channels and the amount of data sent over them must also be defined. The connections in the task graph will represent communication channels, and the amount of data sent will be defined using the communication-to-computation ratio (CCR), a standard metric used in the synthesis of artificial test cases in which the application is given as DAG [43]. The communication time is the time it takes for one task to pass its data to its successor, and computation time is the execution time of a task on a processor. Furthermore, in an MPSoC, data is exchanged between tasks by reading and writing to memory, and depends on memory throughput because memories usually operate at much lower frequencies than processors. Given a defined CCR and the average execution duration and average memory throughput, the amount of data exchanged through the communication channel, the value that is represented by edge weight in DAG, can be calculated using the following equation:

(7) $$amount\_of\_data=\frac{average\_execution\_duration}{average\_memory\_throughput}*CCR$$

For each task graph, A1 to A4, the communication parameters (DAG edge weights) have been generated using the above equation for seven different CCR factors in order to cover a large scale: 0.01, 0.1, 0.5, 1.0, 5.0, 10.0, and 20.0. Additionally, to avoid all tasks exchanging the same amount of data because the average values of execution duration and memory throughput are always the same, the resulting value must be multiplied by a random number from the interval [0.8, 1.2]. The duration of task execution on different processors and memory parameters is defined in the platform model, which will be discussed next.

Platform models in this benchmark are built to correspond to the two basic types of clustered platforms: (1) platforms with homogeneous clusters, each suited for a different purpose (e.g., DSP), and (2) tile-like platforms where each tile contains several processors of different types, but all tiles are the same, as described in Section 1. All models contain 4 clusters, with 4 processors and one memory in each cluster, and one memory used for connecting clusters. Four different types of processors are used to correspond to four types of execution nodes in PSPLib, where each task can require from one to four nodes for its execution: *j301_1*—one node per task, *j305_4*—2 or 3 nodes per task, *j3010_1*—3 to 4 nodes, and *j3014_4*—4 nodes per task. This is converted to a constraint that all processors are not able to execute all tasks from all application models, important for MPSoC. Consequently, each type of processor is able to execute one quarter of tasks from model A1, around half from model A2, three quarters from model A3, and all tasks from model A4. Furthermore, two different types of memories are used, one for connecting elements inside the cluster, and one for connecting clusters. The execution time of each procedure is defined for each processor, and in cases where a certain procedure cannot be executed on a certain processor, the execution time is defined as infinite. Platform parameters such as the memory size and throughput, and the number of read and write ports, are based on the properties of typical embedded development platforms like Zynq [44] and Parallella [3].

**Figure 7:**
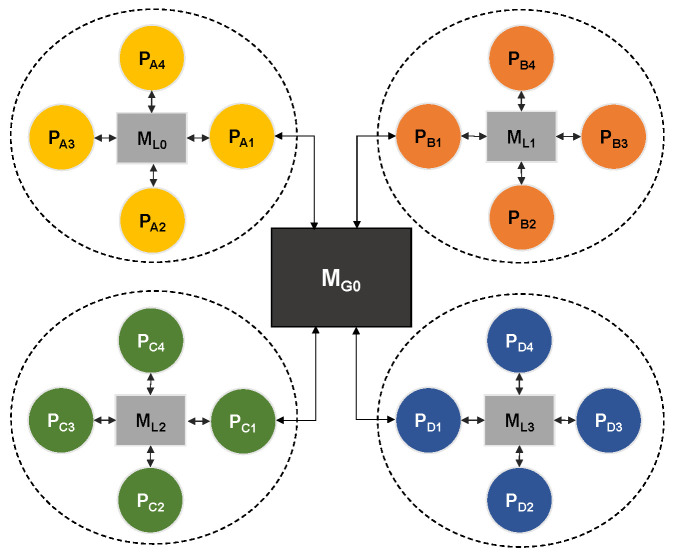
Platform model P1.

In the first model—*P1*, shown in Figure 7, processors of the same type form a cluster. In the figure, the type of processor is indicated by a letter in subscript ($P_{A}$, $P_{B}$, etc.) All processors inside the cluster are connected to one local memory. Local memories in all clusters are of the same type. One memory element ($M_{G0}$) is used to connect all four clusters, and one processor from each cluster is connected to it. In the real world, this model represents a platform where cores of the same type are grouped together (e.g., CPU cores, GPU cores, DSP cores, etc.).

**Figure 8:**
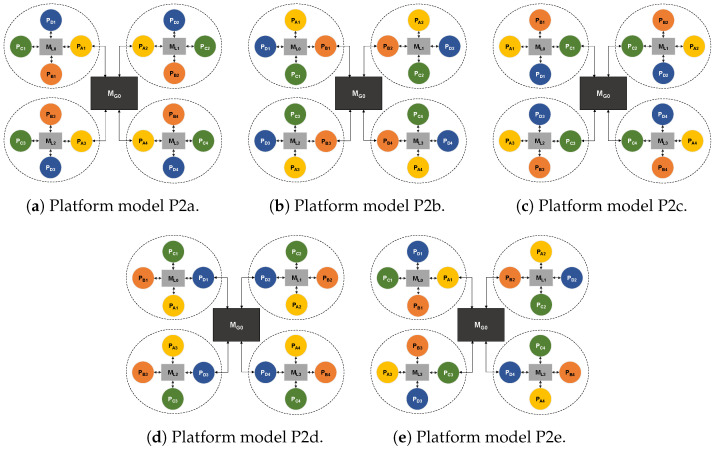
The five sub-models of platform model P2, each one composed of four tile-like clusters.

The *P2* model represents tile-like platforms, and each cluster contains a single instance of each processor type, as illustrated in Figure 8. Again, processors inside the cluster are connected to one local memory, local memories in all clusters are of the same type, and one memory element is used to connect all four clusters. However, for this type of layout, there are multiple ways a cluster can be connected to the central hub (memory). Five sub-models are created: P2a (Figure 8a) to P2e (Figure 8e), depending on which type of processor is connected to the hub. In models P2a to P2d, processors of the same type are connected to the hub ($P_{A}$ in model P2a, $P_{B}$ in model P2b, etc.). In the P2e model, a different processor type is chosen in each cluster, so there are four different types of processors connected to central memory.

In total, 28 application and 6 platform models have been created and used for evaluation with the results presented in the next section. We would also like to point out that platform models are specified using XML and are easily extendable to incorporate more clusters and more elements in each cluster, which would be useful in the future research. All models are publicly available on git repository at https://gitlab.com/Frid/dsesparse (accessed on 10 October 2022).

### 5.2. Experimental Results

All proposed algorithms, along with the SDSE algorithm, have been tested on all combinations of the application and platform models described in the previous section, resulting in over 150 test cases. Basic algorithm parameters such as the population size, stopping criteria, and genetic operators were the same for all algorithms and all test cases, as follows:

- starting population: 100 randomly chosen chromosomes
- algorithm stops after $10^{5}$ generations
- crossover and mutation operators [45]: *1x*, *2x*, *ux*, *sbx*, *spx*, *pcx*, *undx*, *pm* and *um*
- 100 repetitions for each test case.

The algorithms will be evaluated according to two performance metrics. First, they will be evaluated according to the success rate of obtaining feasible solutions. This metric is important as it outlines the probability of each algorithm in obtaining usable solutions. Thus, an algorithm with a low success rate is not acceptable as it has a low probability to obtain feasible solutions, and will thus have to be executed many times. Second, the feasible solutions are then evaluated according to MO performance metrics to analyze the convergence and diversity of the solutions obtained by each algorithm. With these metrics, it is then possible to determine how good the feasible solutions obtained by each algorithm are.

#### 5.2.1. Success Rate

The breakdown of test results across different application and platform models is given in Table 1 and Table 2. In the case of platform P1, where processors of the same type are grouped together, the SDSE and the N-SDSE algorithms are almost completely unable to find feasible solutions. On the other hand, the other four algorithms, which have adjusted the mapping strategy towards a clustered architecture, have a 100% success rate. The situation is more diverse in the case of the P2 platform model, with the tile-like structure, and its different sub-versions. Here, for models P2a to P2d, for which it is always the same type of processor connected to the central memory, neither algorithm has a 100% success rate, and algorithms SDSE and MC-SDSE have almost the same results at around 80% success rate. However, for model P2e, MC-SDSE and MCN-SDSE algorithms, which prioritize the processors around the main memory, are again 100% successful, and the other two cluster-oriented algorithms are not too far behind. The common thing between platform models P1 and P2e is that there is at least one instance of each processor type connected to central memory. Algorithms MC-SDSE and MCN-SDSE will exploit this property directly, as they were designed to do, but other cluster-oriented algorithms (which treat all clusters equally) seem to also be able to benefit from this property. On the other hand, the SDSE algorithm prioritizes processors that have more connections to other processors, and in the case of platform P1, it clashes with the high specialization of processors in all clusters but the central one. Since the SDSE is completely unaware of cluster structure. it will not be able to identify that central one, and hence it gives very bad results. For the case of model P2e, each cluster has different types of processors, so in this case, the SDSE’s prioritization of better connectivity will not cause issues with task executability.

On average, The MC-SDSE algorithm was the most successful and was able to find feasible solutions in 86% of test cases. The least successful was the N-SDSE algorithm which was able to find feasible solutions in only 15% of test cases. The SDSE algorithm, that is not originally designed for sparsely connected clustered platforms, was the second most successful with overall success in 70% of the test cases.

It is also interesting to note that all algorithms that have non-infinite penalization of infeasible solutions (N-SDSE, CN-SDSE, MCN-SDSE) are less successful than their counterparts that give infeasible solutions infinite penalties. The biggest difference is between algorithms SDSE and NSDSE, where the former is the overall second most successful, and the latter is the least successful. At first glance, this is somewhat unexpected since the penalty factor is quite large and should be able to adequately narrow down the search region. However, the penalty factor depends very much on the optimization problem that is being solved, and there is no single method for calculating its value [9]. Here, it is suspected that because infeasible solutions greatly outnumber feasible ones, it is better for the search algorithm to immediately discard infeasible solutions instead of trying to refine them.

In order to understand why the MC-SDSE and MCN-SDSE are not able to achieve 100% success rate for models P2a to P2d, it is necessary to look at the breakdown of test results across application models in Table 2. They both have 100% success for model A4, and MC-SDSE is also 100% successful for A1, but application models A2 and A3 seem problematic for both algorithms. The main reason is probably that when application models A2 and A3 are combined with platform models P2a to P2d, one half to one quarter of the tasks will not be able to execute on processors connected to the central memory which are prioritized by the MC-SDSE and MCN-SDSE algorithms. However, in the case of application models A1 and A4, the strategy of prioritizing the “main cluster” seems beneficial. For the case of application model A4, this is expected, since all tasks can execute on all processors, but for the A1 model, the answer is not so straightforward because it has the highest constraint on the execution (each task can be executed by only one quarter of processors). However, in model A1 the complexity of connections between predecessors and successors is also the lowest and it seems that this is helpful for the MC-SDSE algorithm.

As a closing remark regarding the success rate in finding feasible solutions, we would like to clarify a bit more the meaning of results achieved by different algorithms in relation to real-world implications. The platform model P1, where the cores of the same type are grouped together, represents a type of architecture that is highly prevalent on the market according to the survey in [46], with ARM big.LITTLE [47] architecture as a prominent example, in which the high-performance (big) and high-efficiency (LITTLE) Cortex cores are combined, and serve as the basis for Exynos processors on smartphones. For that type of platform, the four new algorithms are 100% successful, while the SDSE algorithm is almost completely unable to find any feasible solutions (4% success). Furthermore, while in the case of platform P2, only the MC-SDSE algorithm outperforms the SDSE, the new algorithms (apart from N-SDSE) are on average successful in 50 or more percent of cases, which is significantly better than the performance of the SDSE on platform P1 but does call for more research and future improvement.

#### 5.2.2. Multi-Objective Metrics

Since these are all multi-objective algorithms, they have been also compared using the hypervolume (HV) and inverted generational distance (IGD) metrics, as described in Section 4. The detailed results of all algorithms and all test cases are given in Table 3 and Table 4. Empty table cells indicate no feasible solution was found.

**Figure 9:**
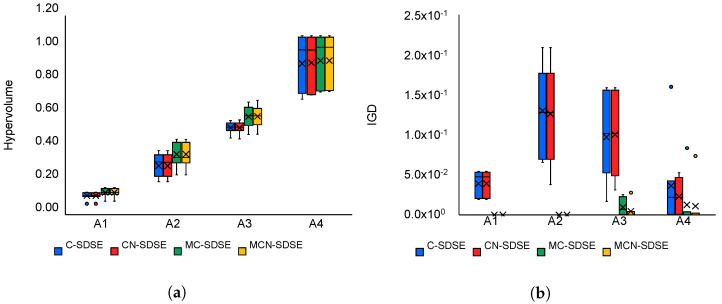
The comparison of the hypervolume (HV)—(**a**), and the inverted generational distance (IGD)—(**b**) metrics of algorithms that base their search on clusters in case of platform model P1.

Comparison of all cluster-based algorithms for platform model P1, according to the HV metric, is illustrated in Figure 9a (higher value is better). It is clear that there is very little difference between them, with MC-SDSE and MCN-SDSE being just slightly better. The achieved value of the HV metric, for all algorithms, mostly depends on the application model: it is lowest for A1 and highest for A4. This is expected because the constraints on execution are the greatest for model A1, and none for model A4, which in turn means, that the solution space is smallest for A1, and largest for A4. The comparison of these algorithms according to IGD metric, presented in Figure 9b, again shows that MC-SDSE and MCN-SDSE perform a little bit better (a lower value is better).

**Figure 10:**
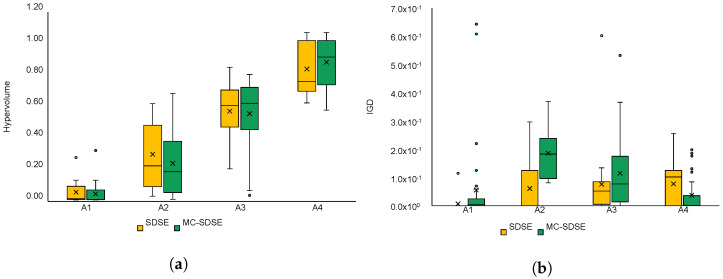
The comparison of the hypervolume (HV)—(**a**), and the inverted generational distance (IGD)—(**b**) metrics of SDSE and MC-SDSE algorithms for platform model P2.

In the case of platform P2 and its various subversions, SDSE and MC-SDSE algorithms have by far the highest success rate in finding feasible solutions, so a more detailed comparison of these two algorithms according to the hypervolume measure is shown for all P2 models in Figure 10a. Here, only test cases where both algorithms have been able to find feasible solutions have been taken into account. The MC-SDSE again outperforms the SDSE, except in the case of application model A1, but the difference is rather small. According to the IGD metric, the SDSE seems to perform better in most cases, but the difference is again very small with the order of magnitude $10^{-2}$.

## 6. Conclusions

In this paper, we have presented five new algorithms for design space exploration of clustered sparsely connected heterogeneous platforms. The algorithms base their search process on the NSGA-II evolutionary meta-heuristic and implement different mapping strategies to cope with a large number of infeasible solutions in the design space.

Because of the lack of existing models for the evaluation of this particular type of platform, we have proposed a new synthetic benchmark. The benchmark encompasses multiple application and platform models, with constraints like specialized types of processors, task concurrency, and precedence constraints.

The algorithms were tested on over 150 test cases and compared with the previously published SDSE algorithm according to three metrics: success rate in finding feasible solutions, HV, and IGD. Overall, four new algorithms (C-SDSE, CN-SDSE, MC-SDSE, and MCN-SDSE) had 100% success in the case of a platform with dedicated clusters, which is today a very widespread embedded multiprocessor architecture. Compared to only 4% success of the SDSE algorithm, this is considered a significant improvement. According to HV and IGD metrics, the MC-SDSE and MCN-SDSE performed slightly better than the others. In the case of tile-like platform layouts, the results were more diverse and neither algorithm had a 100% success rate, but the algorithms that gave priority to processors around the main hub gave better results. Here, the only real issues were combinations of application and platform models in which only one type of processor was connected to the main hub, and those processors were not able to execute all tasks. Furthermore, the results for the tile-like platforms show that algorithms that treat all infeasible solutions equally (i.e., objective values of infeasible solutions to infinity) achieve better results compared to ones that use finite penalties.

The limitations of our study and possible directions for future research are the following. Currently, all application models have a DAG structure, which means that we have not considered applications that have tasks in loops, recursive tasks, or streaming applications. However, we believe that current application models can be easily adapted to include looped and recursive tasks, which is considered a topic for future research. On the other hand, adapting to streaming applications, as a more dynamic environment, would probably require significant adaptation of the mapping and scheduling strategies in the algorithms themselves. As for the platform models, in the case when there is no direct connection between processors that executed two consecutive tasks, a possible solution worth investigating would be to route data through additional processors which would serve as a bridge. This would eliminate infeasible solutions due to lack of connectivity, but it would introduce another level of complexity, and other issues related to throughput and routing impact on effective usage of platform resources would have to be resolved.

A likely first step in our future research would be to add energy consumption as an additional objective. Energy saving, as a part of *green computing*, is currently a relevant subject, especially in the areas like IoT. Since energy consumption depends on the duration and the type of platform elements used, the proposed algorithms should require only minor adjustments. Furthermore, these algorithms should be further evaluated on an even more complex set of sparsely connected platforms where the clusters would significantly vary in size, types of processors they contain, and the level of interconnectivity—from very sparse to almost fully interconnected.

## Figures and Tables

**Table 1:** The detailed overview of success rate of each algorithm for each platform model.

	SDSE	N-SDSE	C-SDSE	CN-SDSE	MC-SDSE	MCN-SDSE
P1	4%	0%	**100%**	**100%**	**100%**	**100%**
P2 (AVG)	**84%**	18%	59%	45%	**84%**	59%
P2a	**89%**	14%	50%	25%	79%	57%
P2b	75%	4%	43%	21%	**79%**	32%
P2c	**82%**	14%	64%	46%	**82%**	50%
P2d	**79%**	0%	46%	25%	**79%**	54%
P2e	93%	57%	93%	79%	**100%**	**100%**
All platforms (AVG)	70%	15%	66%	49%	**86%**	65%

**Table 2:** The detailed overview of success rate of each algorithm for each application model.

	SDSE	N-SDSE	C-SDSE	CN-SDSE	MC-SDSE	MCN-SDSE
A1	86%	0%	81%	19%	**100%**	36%
A2	45%	5%	33%	33%	**62%**	43%
A3	67%	24%	52%	48%	**83%**	**83%**
A4	83%	31%	98%	98%	**100%**	**100%**

**Table 3:** Hypervolume of the Pareto-optimal results obtained by each algorithm for each test case.

		P1						P2a						P2b					
		SDSE	N-SDSE	C-SDSE	CN-SDSE	MC-SDSE	MCN-SDSE	SDSE	N-SDSE	C-SDSE	CN-SDSE	MC-SDSE	MCN-SDSE	SDSE	N-SDSE	C-SDSE	CN-SDSE	MC-SDSE	MCN-SDSE
0.01	A1			1.10 × 10^−1^	1.10 × 10^−1^	1.35 × 10^−1^	1.35 × 10^−1^	1.24 × 10^−2^		1.00 × 10^−2^		1.24 × 10^−2^		1.35 × 10^−1^		1.10 × 10^−1^		1.35 × 10^−1^	
	A2			1.99 × 10^−1^	1.99 × 10^−1^	2.41 × 10^−1^	2.41 × 10^−1^					1.06 × 10^−1^						4.11 × 10^−2^	
	A3			4.63 × 10^−1^	4.57 × 10^−1^	4.84 × 10^−1^	4.85 × 10^−1^	4.26 × 10^−2^										2.67 × 10^−1^	
	A4			7.31 × 10^−1^	7.23 × 10^−1^	7.46 × 10^−1^	7.49 × 10^−1^	7.38 × 10^−1^	6.28 × 10^−1^	6.60 × 10^−1^	6.54 × 10^−1^	6.89 × 10^−1^	6.80 × 10^−1^	7.93 × 10^−1^		7.06 × 10^−1^		7.53 × 10^−1^	7.61 × 10^−1^
0.10	A1	8.12 × 10^−2^		6.62 × 10^−2^	6.62 × 10^−2^	8.12 × 10^−2^	8.12 × 10^−2^	1.24 × 10^−2^		1.00 × 10^−2^		1.24 × 10^−2^		1.26 × 10^−1^				1.24 × 10^−1^	
	A2			3.18 × 10^−1^	3.17 × 10^−1^	3.44 × 10^−1^	3.44 × 10^−1^					2.06 × 10^−1^						3.94 × 10^−2^	
	A3			5.52 × 10^−1^	5.44 × 10^−1^	5.59 × 10^−1^	5.60 × 10^−1^	1.59 × 10^−1^											
	A4			6.96 × 10^−1^	7.24 × 10^−1^	7.39 × 10^−1^	7.42 × 10^−1^	6.98 × 10^−1^	6.99 × 10^−1^	6.24 × 10^−1^	6.19 × 10^−1^	6.42 × 10^−1^	6.45 × 10^−1^	7.61 × 10^−1^		6.46 × 10^−1^	3.48 × 10^−1^	6.89 × 10^−1^	6.88 × 10^−1^
0.50	A1			1.36 × 10^−1^	1.36 × 10^−1^	1.64 × 10^−1^	1.64 × 10^−1^	1.00 × 10^−2^				1.00 × 10^−2^		1.31 × 10^−1^		1.05 × 10^−1^		1.31 × 10^−1^	
	A2			2.31 × 10^−1^	2.31 × 10^−1^	3.13 × 10^−1^	3.13 × 10^−1^	8.83 × 10^−2^				6.27 × 10^−2^		3.95 × 10^−2^					
	A3			5.68 × 10^−1^	5.72 × 10^−1^	5.96 × 10^−1^	5.97 × 10^−1^	3.29 × 10^−1^					1.87 × 10^−1^	2.06 × 10^−1^				6.83 × 10^−2^	4.83 × 10^−2^
	A4			7.91 × 10^−1^	8.03 × 10^−1^	8.38 × 10^−1^	8.35 × 10^−1^	7.07 × 10^−1^		7.79 × 10^−1^	7.83 × 10^−1^	8.54 × 10^−1^	8.51 × 10^−1^	7.19 × 10^−1^		8.40 × 10^−1^	4.26 × 10^−1^	8.69 × 10^−1^	8.70 × 10^−1^
1.00	A1			1.33 × 10^−1^	1.33 × 10^−1^	1.58 × 10^−1^	1.58 × 10^−1^	1.26 × 10^−2^		1.00 × 10^−2^		1.26 × 10^−2^		1.27 × 10^−1^		1.02 × 10^−1^		1.15 × 10^−1^	
	A2			3.61 × 10^−1^	3.61 × 10^−1^	4.36 × 10^−1^	4.36 × 10^−1^	1.00 × 10^−1^				6.53 × 10^−2^	4.26 × 10^−2^						
	A3			5.08 × 10^−1^	5.08 × 10^−1^	5.39 × 10^−1^	5.42 × 10^−1^	3.61 × 10^−1^		7.63 × 10^−2^			2.35 × 10^−1^			3.92 × 10^−2^		1.53 × 10^−2^	
	A4			9.94 × 10^−1^	9.93 × 10^−1^	1.01 × 10^0^	1.01 × 10^0^	7.98 × 10^−1^		9.56 × 10^−1^	9.60 × 10^−1^	9.80 × 10^−1^	9.79 × 10^−1^	6.60 × 10^−1^		8.42 × 10^−1^	3.29 × 10^−1^	8.72 × 10^−1^	8.72 × 10^−1^
5.00	A1			1.15 × 10^−1^	1.15 × 10^−1^	1.26 × 10^−1^	1.26 × 10^−1^	1.00 × 10^−2^				1.00 × 10^−2^		1.33 × 10^−1^				1.33 × 10^−1^	
	A2			3.26 × 10^−1^	3.26 × 10^−1^	4.23 × 10^−1^	4.23 × 10^−1^	2.25 × 10^−1^				5.06 × 10^−2^	5.34 × 10^−2^	1.00 × 10^−2^					
	A3			5.28 × 10^−1^	5.28 × 10^−1^	6.48 × 10^−1^	6.41 × 10^−1^	2.20 × 10^−1^		5.39 × 10^−2^			1.88 × 10^−1^	3.89 × 10^−1^				1.85 × 10^−1^	
	A4			1.02 × 10^0^	1.02 × 10^0^	1.02 × 10^0^	1.02 × 10^0^	7.46 × 10^−1^	7.45 × 10^−1^	1.02 × 10^0^	1.02 × 10^0^	1.02 × 10^0^	1.02 × 10^0^	9.38 × 10^−1^		9.42 × 10^−1^	2.92 × 10^−1^	9.45 × 10^−1^	9.45 × 10^−1^
10.00	A1			1.33 × 10^−1^	1.33 × 10^−1^	1.48 × 10^−1^	1.48 × 10^−1^	7.25 × 10^−2^		6.48 × 10^−2^		7.25 × 10^−2^		8.42 × 10^−2^				8.42 × 10^−2^	
	A2			3.86 × 10^−1^	3.86 × 10^−1^	4.55 × 10^−1^	4.55 × 10^−1^					1.27 × 10^−2^	1.00 × 10^−2^	1.28 × 10^−2^					
	A3			5.53 × 10^−1^	5.53 × 10^−1^	6.79 × 10^−1^	6.89 × 10^−1^	3.56 × 10^−1^					1.36 × 10^−1^					3.11 × 10^−1^	
	A4			1.08 × 10^0^	1.08 × 10^0^	1.08 × 10^0^	1.08 × 10^0^	1.03 × 10^0^	1.03 × 10^0^	1.03 × 10^0^	1.03 × 10^0^	1.03 × 10^0^	1.03 × 10^0^	1.02 × 10^0^	7.09 × 10^−1^	1.02 × 10^0^	7.11 × 10^−1^	1.02 × 10^0^	1.02 × 10^0^
20.00	A1			1.11 × 10^−1^	1.11 × 10^−1^	1.22 × 10^−1^	1.22 × 10^−1^	1.29 × 10^−2^		1.00 × 10^−2^		1.20 × 10^−2^		1.13 × 10^−1^		8.52 × 10^−2^		1.02 × 10^−1^	
	A2			2.43 × 10^−1^	2.43 × 10^−1^	3.40 × 10^−1^	3.40 × 10^−1^	3.30 × 10^−2^				1.33 × 10^−2^	1.33 × 10^−2^	1.00 × 10^−2^					
	A3			5.07 × 10^−1^	5.07 × 10^−1^	6.40 × 10^−1^	6.38 × 10^−1^	4.01 × 10^−1^				3.86 × 10^−2^	1.71 × 10^−1^	4.96 × 10^−1^				1.71 × 10^−1^	3.86 × 10^−2^
	A4			1.07 × 10^0^	1.07 × 10^0^	1.07 × 10^0^	1.07 × 10^0^	1.06 × 10^0^	1.06 × 10^0^	1.06 × 10^0^	1.06 × 10^0^	1.06 × 10^0^	1.06 × 10^0^	1.07 × 10^0^		1.07 × 10^0^	5.18 × 10^−1^	1.07 × 10^0^	1.07 × 10^0^
		P2c						P2d						P2e					
		SDSE	N-SDSE	C-SDSE	CN-SDSE	MC-SDSE	MCN-SDSE	SDSE	N-SDSE	C-SDSE	CN-SDSE	MC-SDSE	MCN-SDSE	SDSE	N-SDSE	C-SDSE	CN-SDSE	MC-SDSE	MCN-SDSE
0.01	A1	1.00 × 10^−2^		1.93 × 10^−2^		2.15 × 10^−2^		1.24 × 10^−2^		1.00 × 10^−2^		1.24 × 10^−2^		4.87 × 10^−2^		3.95 × 10^−2^		4.78 × 10^−2^	4.78 × 10^−2^
	A2	1.00 × 10^−2^													3.47 × 10^−1^	4.05 × 10^−1^	3.91 × 10^−1^	4.07 × 10^−1^	4.07 × 10^−1^
	A3	6.29 × 10^−1^		6.50 × 10^−1^	6.51 × 10^−1^	6.47 × 10^−1^	6.90 × 10^−1^	5.89 × 10^−1^				6.38 × 10^−1^	6.56 × 10^−1^	5.64 × 10^−1^	5.32 × 10^−1^	5.94 × 10^−1^	5.96 × 10^−1^	5.95 × 10^−1^	5.96 × 10^−1^
	A4	6.63 × 10^−1^		3.12 × 10^−1^	3.67 × 10^−1^	5.79 × 10^−1^	5.97 × 10^−1^	7.06 × 10^−1^		2.95 × 10^−1^	3.93 × 10^−1^	6.23 × 10^−1^	6.28 × 10^−1^	6.48 × 10^−1^	5.65 × 10^−1^	6.95 × 10^−1^	6.94 × 10^−1^	7.09 × 10^−1^	7.07 × 10^−1^
0.10	A1	1.24 × 10^−2^				1.21 × 10^−2^		1.24 × 10^−2^		1.00 × 10^−2^		1.21 × 10^−2^		5.63 × 10^−2^		4.72 × 10^−2^		4.80 × 10^−2^	4.80 × 10^−2^
	A2					6.14 × 10^−2^						1.00 × 10^−2^		2.15 × 10^−1^		2.03 × 10^−1^	1.81 × 10^−1^	1.88 × 10^−1^	1.89 × 10^−1^
	A3	2.23 × 10^−1^	6.47 × 10^−1^		6.36 × 10^−1^	6.19 × 10^−1^	6.93 × 10^−1^					6.02 × 10^−1^	5.89 × 10^−1^	6.39 × 10^−1^	5.16 × 10^−1^	6.26 × 10^−1^	6.26 × 10^−1^	6.23 × 10^−1^	6.27 × 10^−1^
	A4	7.00 × 10^−1^		5.93 × 10^−1^	3.56 × 10^−1^	6.46 × 10^−1^	6.92 × 10^−1^	7.48 × 10^−1^		7.10 × 10^−1^	4.61 × 10^−1^	7.58 × 10^−1^	6.96 × 10^−1^	6.39 × 10^−1^	5.47 × 10^−1^	6.52 × 10^−1^	6.53 × 10^−1^	6.74 × 10^−1^	6.80 × 10^−1^
0.50	A1	1.24 × 10^−2^		1.00 × 10^−2^		1.06 × 10^−2^		1.24 × 10^−2^		1.00 × 10^−2^		1.08 × 10^−2^		9.61 × 10^−2^		8.80 × 10^−2^		4.80 × 10^−2^	4.80 × 10^−2^
	A2	4.21 × 10^−2^						1.91 × 10^−2^						5.01 × 10^−1^		4.02 × 10^−1^	4.10 × 10^−1^	4.01 × 10^−1^	4.00 × 10^−1^
	A3	5.69 × 10^−1^	5.67 × 10^−1^	5.86 × 10^−1^	5.89 × 10^−1^	4.79 × 10^−1^	6.43 × 10^−1^	6.30 × 10^−1^				6.27 × 10^−1^	6.28 × 10^−1^	6.29 × 10^−1^	6.14 × 10^−1^	5.39 × 10^−1^	5.37 × 10^−1^	5.92 × 10^−1^	5.36 × 10^−1^
	A4	6.86 × 10^−1^		7.94 × 10^−1^	3.76 × 10^−1^	8.30 × 10^−1^	8.31 × 10^−1^	6.25 × 10^−1^		5.71 × 10^−1^	3.62 × 10^−1^	7.91 × 10^−1^	7.91 × 10^−1^	6.64 × 10^−1^	5.94 × 10^−1^	7.10 × 10^−1^	7.10 × 10^−1^	7.39 × 10^−1^	7.37 × 10^−1^
1.00	A1	1.27 × 10^−2^		1.01 × 10^−2^		1.00 × 10^−2^		1.26 × 10^−2^		1.00 × 10^−2^		1.03 × 10^−2^		3.20 × 10^−2^				1.00 × 10^−2^	1.00 × 10^−2^
	A2													4.45 × 10^−1^		3.49 × 10^−1^	3.49 × 10^−1^	3.49 × 10^−1^	3.49 × 10^−1^
	A3	7.15 × 10^−1^		6.44 × 10^−1^	6.46 × 10^−1^	7.19 × 10^−1^	7.19 × 10^−1^	3.57 × 10^−1^				3.82 × 10^−1^	3.88 × 10^−1^	5.11 × 10^−1^	5.08 × 10^−1^	5.34 × 10^−1^	5.31 × 10^−1^	6.07 × 10^−1^	6.07 × 10^−1^
	A4	7.25 × 10^−1^	7.12 × 10^−1^	7.03 × 10^−1^	4.46 × 10^−1^	9.11 × 10^−1^	9.16 × 10^−1^	6.70 × 10^−1^		1.86 × 10^−1^	4.17 × 10^−1^	9.16 × 10^−1^	9.14 × 10^−1^	9.39 × 10^−1^	9.19 × 10^−1^	9.13 × 10^−1^	9.10 × 10^−1^	9.47 × 10^−1^	9.46 × 10^−1^
5.00	A1	1.70 × 10^−2^		1.46 × 10^−2^		1.00 × 10^−2^		1.91 × 10^−2^		1.68 × 10^−2^		1.32 × 10^−2^		2.79 × 10^−1^		2.75 × 10^−1^		3.23 × 10^−1^	4.49 × 10^−2^
	A2					1.00 × 10^−2^		1.00 × 10^−2^						6.20 × 10^−1^	5.10 × 10^−1^	6.58 × 10^−1^	6.97 × 10^−1^	6.84 × 10^−1^	3.32 × 10^−1^
	A3					5.60 × 10^−1^	5.63 × 10^−1^	7.12 × 10^−1^				7.38 × 10^−1^	7.40 × 10^−1^	7.75 × 10^−1^	5.39 × 10^−1^	6.29 × 10^−1^	6.12 × 10^−1^	8.02 × 10^−1^	6.76 × 10^−1^
	A4	1.01 × 10^0^		1.01 × 10^0^	4.69 × 10^−1^	1.02 × 10^0^	1.02 × 10^0^	1.02 × 10^0^			4.90 × 10^−1^	1.02 × 10^0^	1.02 × 10^0^	9.22 × 10^−1^	9.22 × 10^−1^	9.25 × 10^−1^	9.25 × 10^−1^	9.77 × 10^−1^	9.26 × 10^−1^
10.00	A1	1.69 × 10^−2^		1.48 × 10^−2^		1.48 × 10^−2^		1.57 × 10^−2^		1.36 × 10^−2^		1.36 × 10^−2^	1.00 × 10^−2^	7.08 × 10^−2^				1.00 × 10^−2^	1.00 × 10^−2^
	A2	4.24 × 10^−2^												4.65 × 10^−1^		3.49 × 10^−1^	3.62 × 10^−1^	3.62 × 10^−1^	3.62 × 10^−1^
	A3	7.05 × 10^−1^	8.64 × 10^−1^	7.04 × 10^−1^	7.05 × 10^−1^	8.05 × 10^−1^	8.05 × 10^−1^					4.65 × 10^−1^	4.65 × 10^−1^	7.51 × 10^−1^	6.30 × 10^−1^	6.05 × 10^−1^	6.05 × 10^−1^	6.23 × 10^−1^	6.30 × 10^−1^
	A4	1.07 × 10^0^		1.07 × 10^0^	5.56 × 10^−1^	1.07 × 10^0^	1.07 × 10^0^	1.07 × 10^0^		2.73 × 10^−1^	5.22 × 10^−1^	1.06 × 10^0^	1.06 × 10^0^	9.89 × 10^−1^	9.87 × 10^−1^	9.89 × 10^−1^	9.89 × 10^−1^	9.89 × 10^−1^	9.89 × 10^−1^
20.00	A1	1.29 × 10^−2^		1.00 × 10^−2^		1.17 × 10^−2^		1.20 × 10^−2^		1.00 × 10^−2^		1.20 × 10^−2^		2.92 × 10^−1^		2.88 × 10^−1^	7.42 × 10^−2^	4.41 × 10^−2^	4.41 × 10^−2^
	A2							1.00 × 10^−2^								3.76 × 10^−1^	3.65 × 10^−1^	3.55 × 10^−1^	3.55 × 10^−1^
	A3	7.03 × 10^−1^		6.99 × 10^−1^	7.00 × 10^−1^	8.01 × 10^−1^	8.02 × 10^−1^	5.55 × 10^−1^				6.89 × 10^−1^	6.89 × 10^−1^	8.51 × 10^−1^	8.51 × 10^−1^	7.29 × 10^−1^	7.24 × 10^−1^	7.91 × 10^−1^	7.96 × 10^−1^
	A4	1.06 × 10^0^		1.06 × 10^0^	4.78 × 10^−1^	1.06 × 10^0^	1.06 × 10^0^	1.04 × 10^0^		1.04 × 10^0^	3.51 × 10^−1^	1.04 × 10^0^	1.04 × 10^0^	1.05 × 10^0^	1.05 × 10^0^	1.05 × 10^0^	1.05 × 10^0^	1.05 × 10^0^	1.05 × 10^0^

**Table 4:** IGD values of the Pareto-optimal results obtained by each algorithm for each test case.

		P1						P2a						P2b					
		SDSE	N-SDSE	C-SDSE	CN-SDSE	MC-SDSE	MCN-SDSE	SDSE	N-SDSE	C-SDSE	CN-SDSE	MC-SDSE	MCN-SDSE	SDSE	N-SDSE	C-SDSE	CN-SDSE	MC-SDSE	MCN-SDSE
0.01	A1			5.35 × 10^−2^	5.35 × 10^−2^	0.00 × 10^0^	0.00 × 10^0^	0.00 × 10^0^		2.38 × 10^−2^		0.00 × 10^0^		0.00 × 10^0^		5.37 × 10^−2^		0.00 × 10^0^	
	A2			6.95 × 10^−2^	6.95 × 10^−2^	0.00 × 10^0^	0.00 × 10^0^					0.00 × 10^0^						0.00 × 10^0^	
	A3			7.87 × 10^−2^	8.98 × 10^−2^	1.96 × 10^−3^	0.00 × 10^0^	0.00 × 10^0^										0.00 × 10^0^	
	A4			2.82 × 10^−2^	4.68 × 10^−2^	3.84 × 10^−3^	3.12 × 10^−4^	0.00 × 10^0^	1.19 × 10^−1^	1.02 × 10^−1^	1.05 × 10^−1^	8.43 × 10^−2^	8.62 × 10^−2^	1.60 × 10^−1^		6.09 × 10^−2^		1.98 × 10^−1^	1.97 × 10^−1^
0.10	A1	0.00 × 10^0^		5.25 × 10^−2^	5.25 × 10^−2^	0.00 × 10^0^	0.00 × 10^0^	0.00 × 10^0^		2.40 × 10^−2^		0.00 × 10^0^		0.00 × 10^0^				3.51 × 10^−3^	
	A2			6.56 × 10^−2^	3.79 × 10^−2^	0.00 × 10^0^	0.00 × 10^0^					0.00 × 10^0^						0.00 × 10^0^	
	A3			1.68 × 10^−2^	3.14 × 10^−2^	4.87 × 10^−4^	0.00 × 10^0^	0.00 × 10^0^											
	A4			1.60 × 10^−1^	5.28 × 10^−2^	8.35 × 10^−2^	7.37 × 10^−2^	1.51 × 10^−1^	1.50 × 10^−1^	2.10 × 10^−1^	7.86 × 10^−2^	1.86 × 10^−1^	1.93 × 10^−1^	2.81 × 10^−3^		6.70 × 10^−2^	3.57 × 10^−1^	1.92 × 10^−1^	1.95 × 10^−1^
0.50	A1			5.44 × 10^−2^	5.44 × 10^−2^	0.00 × 10^0^	0.00 × 10^0^	0.00 × 10^0^				0.00 × 10^0^		0.00 × 10^0^		5.55 × 10^−2^		0.00 × 10^0^	
	A2			1.28 × 10^−1^	1.28 × 10^−1^	0.00 × 10^0^	0.00 × 10^0^	0.00 × 10^0^				8.59 × 10^−2^		0.00 × 10^0^					
	A3			5.27 × 10^−2^	4.89 × 10^−2^	2.27 × 10^−2^	0.00 × 10^0^	0.00 × 10^0^					2.09 × 10^−1^	0.00 × 10^0^				1.68 × 10^−1^	2.28 × 10^−1^
	A4			4.25 × 10^−2^	3.60 × 10^−2^	5.35 × 10^−4^	2.10 × 10^−3^	1.12 × 10^−1^		6.57 × 10^−2^	6.57 × 10^−2^	0.00 × 10^0^	6.54 × 10^−3^	1.08 × 10^−1^		4.65 × 10^−2^	2.81 × 10^−1^	2.50 × 10^−2^	0.00 × 10^0^
1.00	A1			4.75 × 10^−2^	4.75 × 10^−2^	0.00 × 10^0^	0.00 × 10^0^	0.00 × 10^0^		2.55 × 10^−2^		0.00 × 10^0^		0.00 × 10^0^		5.49 × 10^−2^		2.70 × 10^−2^	
	A2			2.09 × 10^−1^	2.09 × 10^−1^	0.00 × 10^0^	0.00 × 10^0^	0.00 × 10^0^				1.07 × 10^−1^	1.60 × 10^−1^						
	A3			1.02 × 10^−1^	1.02 × 10^−1^	2.51 × 10^−2^	0.00 × 10^0^	0.00 × 10^0^		3.63 × 10^−1^			1.67 × 10^−1^			1.01 × 10^−1^		1.01 × 10^−1^	
	A4			2.18 × 10^−2^	2.18 × 10^−2^	0.00 × 10^0^	0.00 × 10^0^	7.21 × 10^−2^		4.66 × 10^−2^	3.23 × 10^−2^	6.27 × 10^−4^	1.37 × 10^−3^	1.55 × 10^−1^		2.32 × 10^−2^	4.29 × 10^−1^	0.00 × 10^0^	0.00 × 10^0^
5.00	A1			2.04 × 10^−2^	2.04 × 10^−2^	0.00 × 10^0^	0.00 × 10^0^	0.00 × 10^0^				0.00 × 10^0^		0.00 × 10^0^				0.00 × 10^0^	
	A2			1.51 × 10^−1^	1.51 × 10^−1^	0.00 × 10^0^	0.00 × 10^0^	0.00 × 10^0^				3.69 × 10^−1^	3.74 × 10^−1^	0.00 × 10^0^					
	A3			1.56 × 10^−1^	1.56 × 10^−1^	6.05E-05	4.16 × 10^−3^	1.98 × 10^−1^		3.83 × 10^−1^			5.25 × 10^−2^	0.00 × 10^0^				2.57 × 10^−1^	
	A4			1.31 × 10^−3^	1.31 × 10^−3^	0.00 × 10^0^	0.00 × 10^0^	2.50 × 10^−1^	2.50 × 10^−1^	0.00 × 10^0^	0.00 × 10^0^	0.00 × 10^0^	0.00 × 10^0^	6.91 × 10^−3^		2.62 × 10^−3^	6.17 × 10^−1^	0.00 × 10^0^	0.00 × 10^0^
10.00	A1			2.61 × 10^−2^	2.61 × 10^−2^	0.00 × 10^0^	0.00 × 10^0^	0.00 × 10^0^		1.99 × 10^−2^		0.00 × 10^0^		0.00 × 10^0^				0.00 × 10^0^	
	A2			1.12 × 10^−1^	1.12 × 10^−1^	0.00 × 10^0^	0.00 × 10^0^					0.00 × 10^0^	2.72 × 10^−2^	0.00 × 10^0^					
	A3			1.59 × 10^−1^	1.59 × 10^−1^	9.27 × 10^−3^	0.00 × 10^0^	0.00 × 10^0^					2.84 × 10^−1^					0.00 × 10^0^	
	A4			5.51 × 10^−4^	5.51 × 10^−4^	0.00 × 10^0^	0.00 × 10^0^	0.00 × 10^0^	0.00 × 10^0^	0.00 × 10^0^	0.00 × 10^0^	0.00 × 10^0^	0.00 × 10^0^	3.01 × 10^−3^	2.60 × 10^−1^	1.14 × 10^−3^	2.60 × 10^−1^	0.00 × 10^0^	0.00 × 10^0^
20.00	A1			1.94 × 10^−2^	1.94 × 10^−2^	0.00 × 10^0^	0.00 × 10^0^	0.00 × 10^0^		2.90 × 10^−2^		9.08 × 10^−3^		0.00 × 10^0^		5.96 × 10^−2^		2.43 × 10^−2^	
	A2			1.77 × 10^−1^	1.77 × 10^−1^	0.00 × 10^0^	0.00 × 10^0^	0.00 × 10^0^				1.97 × 10^−1^	1.97 × 10^−1^	0.00 × 10^0^					
	A3			1.15 × 10^−1^	1.15 × 10^−1^	6.82 × 10^−3^	2.78 × 10^−2^	0.00 × 10^0^				5.32 × 10^−1^	2.63 × 10^−1^	0.00 × 10^0^				3.66 × 10^−1^	6.72 × 10^−1^
	A4			3.69 × 10^−4^	3.69 × 10^−4^	0.00 × 10^0^	0.00 × 10^0^	0.00 × 10^0^		0.00 × 10^0^	0.00 × 10^0^	0.00 × 10^0^	0.00 × 10^0^	3.03 × 10^−4^		3.03 × 10^−4^	4.66 × 10^−1^	0.00 × 10^0^	0.00 × 10^0^
		P2c						P2d						P2e					
		SDSE	N-SDSE	C-SDSE	CN-SDSE	MC-SDSE	MCN-SDSE	SDSE	N-SDSE	C-SDSE	CN-SDSE	MC-SDSE	MCN-SDSE	SDSE	N-SDSE	C-SDSE	CN-SDSE	MC-SDSE	MCN-SDSE
0.01	A1	1.15 × 10^−1^		2.13 × 10^−2^		0.00 × 10^0^		0.00 × 10^0^		1.00 × 10^−2^		3.31 × 10^−4^		0.00 × 10^0^		2.38 × 10^−2^		2.41 × 10^−3^	2.41 × 10^−3^
	A2	0.00 × 10^0^													1.62 × 10^−1^	7.35 × 10^−3^	3.13 × 10^−2^	1.00 × 10^−1^	1.00 × 10^−1^
	A3	1.32 × 10^−1^		5.22 × 10^−2^		6.11 × 10^−2^	6.99 × 10^−2^	7.10 × 10^−2^				2.16 × 10^−2^	1.40 × 10^−2^	3.84 × 10^−2^	1.03 × 10^−1^	3.29 × 10^−3^	2.00 × 10^−3^	2.97 × 10^−3^	9.71 × 10^−4^
	A4	0.00 × 10^0^		3.48 × 10^−1^	2.53 × 10^−1^	1.31 × 10^−1^	1.12 × 10^−1^	0.00 × 10^0^		3.82 × 10^−1^	3.05 × 10^−1^	1.19 × 10^−1^	1.20 × 10^−1^	1.04 × 10^−1^	1.58 × 10^−1^	3.37 × 10^−2^	3.23 × 10^−2^	1.12 × 10^−3^	3.80 × 10^−3^
0.10	A1	0.00 × 10^0^				3.44 × 10^−3^		0.00 × 10^0^		2.40 × 10^−2^		3.03 × 10^−3^		0.00 × 10^0^		2.35 × 10^−2^		2.16 × 10^−2^	2.16 × 10^−2^
	A2					0.00 × 10^0^						0.00 × 10^0^		2.50 × 10^−1^		2.79 × 10^−2^	8.97 × 10^−2^	8.09 × 10^−2^	7.68 × 10^−2^
	A3	6.02 × 10^−1^	4.70 × 10^−2^		6.66 × 10^−2^	8.82 × 10^−2^	1.61 × 10^−2^					1.04 × 10^−3^	1.74 × 10^−2^	2.21 × 10^−2^	1.64 × 10^−1^	8.00 × 10^−2^	7.85 × 10^−2^	7.98 × 10^−2^	7.79 × 10^−2^
	A4	1.22 × 10^−1^		1.96 × 10^−1^	3.52 × 10^−1^	1.76 × 10^−1^	6.43 × 10^−2^	1.19 × 10^−1^		9.74 × 10^−2^	3.10 × 10^−1^	7.24 × 10^−2^	1.59 × 10^−1^	1.18 × 10^−1^	1.47 × 10^−1^	6.26 × 10^−2^	6.29 × 10^−2^	3.58 × 10^−2^	6.13 × 10^−4^
0.50	A1	0.00 × 10^0^		2.42 × 10^−2^		1.82 × 10^−2^		0.00 × 10^0^		2.42 × 10^−2^		1.62 × 10^−2^		0.00 × 10^0^		2.11 × 10^−2^		1.25 × 10^−1^	1.25 × 10^−1^
	A2	0.00 × 10^0^						0.00 × 10^0^						0.00 × 10^0^		1.56 × 10^−1^	1.44 × 10^−1^	1.83 × 10^−1^	1.83 × 10^−1^
	A3	7.80 × 10^−2^	9.37 × 10^−2^	6.54 × 10^−2^	6.39 × 10^−2^	1.84 × 10^−1^	7.88 × 10^−3^	6.93 × 10^−2^				1.63 × 10^−2^	1.90 × 10^−2^	6.10 × 10^−3^	3.56 × 10^−2^	7.16 × 10^−2^	7.16 × 10^−2^	6.35 × 10^−2^	6.52 × 10^−2^
	A4	8.12 × 10^−2^		1.06 × 10^−1^	3.33 × 10^−1^	3.04 × 10^−2^	3.06 × 10^−2^	1.69 × 10^−1^		2.08 × 10^−1^	3.51 × 10^−1^	0.00 × 10^0^	0.00 × 10^0^	1.08 × 10^−1^	1.69 × 10^−1^	5.81 × 10^−2^	5.78 × 10^−2^	2.64 × 10^−2^	2.64 × 10^−2^
1.00	A1	0.00 × 10^0^		2.55 × 10^−2^		2.70 × 10^−2^		0.00 × 10^0^		2.55 × 10^−2^		2.29 × 10^−2^		0.00 × 10^0^				2.20 × 10^−1^	2.20 × 10^−1^
	A2													0.00 × 10^0^		1.75 × 10^−1^	1.75 × 10^−1^	1.75 × 10^−1^	1.75 × 10^−1^
	A3	2.55 × 10^−2^		4.98 × 10^−2^	4.98 × 10^−2^	5.86 × 10^−3^	1.51 × 10^−3^	6.82 × 10^−2^				2.87 × 10^−2^	1.47 × 10^−2^	1.19 × 10^−1^	7.09 × 10^−2^	6.47 × 10^−2^	6.73 × 10^−2^	2.00 × 10^−1^	2.01 × 10^−1^
	A4	1.27 × 10^−1^	1.30 × 10^−1^	1.33 × 10^−1^	3.53 × 10^−1^	3.38 × 10^−3^	0.00 × 10^0^	1.28 × 10^−1^		6.65 × 10^−1^	3.27 × 10^−1^	2.69 × 10^−3^	1.57 × 10^−3^	9.49 × 10^−3^	2.75 × 10^−2^	3.60 × 10^−2^	3.76 × 10^−2^	0.00 × 10^0^	7.41 × 10^−2^
5.00	A1	0.00 × 10^0^		2.37 × 10^−2^		6.95 × 10^−2^		0.00 × 10^0^		2.32 × 10^−2^		5.88 × 10^−2^		1.15 × 10^−1^		1.25 × 10^−1^		0.00 × 10^0^	7.21 × 10^−1^
	A2					0.00 × 10^0^		0.00 × 10^0^						2.97 × 10^−1^	6.92 × 10^−2^	4.77 × 10^−2^	1.56 × 10^−2^	2.46 × 10^−1^	5.73 × 10^−1^
	A3					5.30 × 10^−3^	7.23 × 10^−3^	4.24 × 10^−2^				5.90 × 10^−3^	0.00 × 10^0^	3.01 × 10^−2^	2.41 × 10^−1^	1.53 × 10^−1^	1.64 × 10^−1^	0.00 × 10^0^	1.65 × 10^−1^
	A4	1.15 × 10^−1^		1.15 × 10^−1^	3.86 × 10^−1^	0.00 × 10^0^	0.00 × 10^0^	0.00 × 10^0^			4.71 × 10^−1^	2.36 × 10^−3^	2.36 × 10^−3^	2.56 × 10^−1^	2.56 × 10^−1^	1.11 × 10^−1^	1.11 × 10^−1^	0.00 × 10^0^	2.55 × 10^−1^
10.00	A1	0.00 × 10^0^		2.03 × 10^−2^		2.03 × 10^−2^		0.00 × 10^0^		2.06 × 10^−2^		2.06 × 10^−2^	5.67 × 10^−2^	0.00 × 10^0^				6.08 × 10^−1^	6.08 × 10^−1^
	A2	0.00 × 10^0^												0.00 × 10^0^		2.83 × 10^−1^	2.30 × 10^−1^	2.30 × 10^−1^	2.30 × 10^−1^
	A3	8.89 × 10^−2^	0.00 × 10^0^	9.01 × 10^−2^	9.03 × 10^−2^	7.46 × 10^−2^	7.46 × 10^−2^					0.00 × 10^0^	0.00 × 10^0^	8.37 × 10^−2^	7.99 × 10^−2^	1.04 × 10^−1^	1.19 × 10^−1^	1.72 × 10^−1^	1.74 × 10^−1^
	A4	1.02 × 10^−1^		1.02 × 10^−1^	3.41 × 10^−1^	1.07 × 10^0^	1.07 × 10^0^	0.00 × 10^0^		7.69 × 10^−1^	4.62 × 10^−1^	8.49 × 10^−4^	8.49 × 10^−4^	0.00 × 10^0^	1.98 × 10^−3^	1.98 × 10^−3^	1.98 × 10^−3^	0.00 × 10^0^	0.00 × 10^0^
20.00	A1	0.00 × 10^0^		2.90 × 10^−2^		1.19 × 10^−2^		0.00 × 10^0^		2.01 × 10^−2^		0.00 × 10^0^		0.00 × 10^0^		1.02 × 10^−2^	2.86 × 10^−1^	6.43 × 10^−1^	6.43 × 10^−1^
	A2							0.00 × 10^0^								8.80 × 10^−4^	2.04 × 10^−2^	6.64 × 10^−2^	6.64 × 10^−2^
	A3	1.34 × 10^−1^		3.91 × 10^−2^	3.89 × 10^−2^	1.01 × 10^−1^	0.00 × 10^0^	6.13 × 10^−2^				0.00 × 10^0^	0.00 × 10^0^	5.56 × 10^−4^	0.00 × 10^0^	5.31 × 10^−2^	7.75 × 10^−2^	1.02 × 10^−1^	8.87 × 10^−2^
	A4	1.25 × 10^−1^		1.25 × 10^−1^	3.87 × 10^−1^	0.00 × 10^0^	0.00 × 10^0^	0.00 × 10^0^		0.00 × 10^0^	5.97 × 10^−1^	6.14 × 10^−4^	6.14 × 10^−4^	5.49 × 10^−4^	5.49 × 10^−4^	5.49 × 10^−4^	5.49 × 10^−4^	0.00 × 10^0^	0.00 × 10^0^

## Data Availability

The algorithm source code and system models are publicly available on git repository at https://gitlab.com/Frid/dsesparse (accessed on 10 October 2022).

## Abbreviations

The following abbreviations are used in this manuscript:

NSGA-II	Non-dominated Sorting Genetic Algorithm II
DSE	Design space exploration
SDSE	Single-phased Design Space Exploration algorithm
HV	Hypervolume
IGD	Inverted Generational Distance
POF	Pareto-optimal Front
CCR	Communication-to-Computation Ratio
